# Supplementary material for: A sparse quantized hopfield network for online-continual memory
Source: Nat Commun. 2024 May 2;15:3722. doi: 10.1038/s41467-024-46976-4 (PMC11065890; doi:10.1038/s41467-024-46976-4)
Supplement: Supplementary file 1 — Supplementary Information [file 41467_2024_46976_MOESM1_ESM.pdf]

A Sparse Quantized Hopfield Network for Online-Continual Memory

1 Supplementary Material

1.1 Supplementary Note 1: Derivation of Energy

Consider a directed tree-structured acyclic graph (DAG) where each hidden node represents a discrete random variable. The joint probability of the nodes values, given the learned parameters, is the product of the conditional probabilities of each node given the values of its parent nodes:

$$p(h_0^*, h_1^*, ...h_L^*) = \prod_{l=0}^{L} p(h_l^* | pa_l^t), \tag{1}$$

where pa_l refers to the values of the parents of nodes l and h_l^* is the integer value assigned to node l. Here we show our novel energy function can be interpreted as an approximation to this joint distribution, when we place a prior distribution over parameters, representing uncertainty. As we explain, adding this prior allows us to treat learning as Bayesian inference, which is highly useful for online learning.

Let $M_{pa_l,l}$ be the matrix of learned conditional probabilities over node l given its parent. The values of the parent nodes are discrete integer values represented, in SQHN networks, by the one-hot $h_{pa_l}^*$. The conditional distribution according to the learned matrix is $p_l = M_{pa_l,l} h_{pa_l}^*$, which is equivalent to the column of M_{pa_l} indexed by $h_{pa_l}^*$. However, p_l does not take into account the uncertainty over our parameters. Accounting for uncertainty is important especially early in online training, since early in training parameters have been updated using only a tiny fraction of the data set and are likely therefore to be biased.

A common method to represent uncertainty over parameters is to treat parameters as a random variable and place a prior distribution over it. Learning in this regime amounts to performing Bayesian inference, where the maximum likelihood (learned) parameters are combined with the prior distribution. The typical prior distribution over parameters for discrete graphical models, like Bayesian networks, are Dirichlet priors, which are the conjugate prior for discrete (e.g., categorical and multinomial) distributions. Details of about this prior can be found in [5]. Here we simply point out that the most common Dirichlet prior is the uniform distribution. If node l is a discrete distribution then the distribution the represents no prior knowledge is the uniform distribution.

There are several ways to place this prior over parameters. First, a prior distribution may be place over each individual column of $M_{pa_l,l}$, where each column represents the conditional distribution for a different parent node value. In this case, uncertainty about the distribution encoded in each column of $M_{pa_l,l}$ is represented separately, and may differ between columns. We use a simpler approach, which is to have one measure of uncertainty over $M_{pa_l,l}$ as a whole. Let's call θ^0 the parameters that set each column of each matrix equal to the uniform distribution. In particular, if we assume that the parent of l is assigned value j, we compute the conditional probability distribution as

$$p(h_l^* | pa_l, \theta^0, \alpha) = \frac{\alpha \frac{1}{J_l} + t p(h_l^* | pa_l)}{t + \alpha} = \frac{\epsilon}{J_l} + (1 - \epsilon) p(h_l^* | pa_l), \tag{2}$$

where t is the total number of data points observed so far and $\epsilon = \frac{\alpha}{t+\alpha}$. The value J_l is the number of values node l can take and thus $\frac{1}{J_l}$ represents the prior (uniform) probability over child node values. The probability $p(h_l^* | pa_l)$ is the learned conditional probability of h_l^* generated by $M_{pa_l,l}$. The conditional probability at iteration t then is a weighted average between the uniform distribution and the learned distribution generated by $M_{pa_l,l}$. The learned distribution is weighted increasingly heavily as the number of data points observed, t, increases. The weighting also depends on the hyper-parameter α: the larger α is the more heavily the uniform distribution is weighted and the slower its influence will decay.

Taking the joint of these conditionals we get

$$p(h_0^*, h_1^*, ..., h_L^*) = \prod_{l=0}^{L} p(h_l^*|pa_l, \theta^0, \alpha) = \prod_{l=0}^{L} (\frac{\epsilon}{J_l} + (1-\epsilon)p(h_l^*|pa_l)). \tag{3}$$

We derive an energy from this expression by approximating the maximization of the joint distribution. It is approximated by expanding the product and removing certain terms that are guaranteed to be small relative to other terms under certain assumptions and by removing constants which do not affect the local maximum of the joint. To simplify notation, without loss of generality, assume all nodes have the same number of possible values J. Further, let $g = \frac{\epsilon}{J}$ and $p(h_l^*|pa_l) = p_l$ and L be the number of nodes. Now if we expand the equation above we get a combination of sums and products of probabilities:

$$\begin{aligned}\prod_{l=0}^{L}(g + (1-\epsilon)p_l) &= g^L + g^{L-1}(1-\epsilon)(\sum_{l=1}^{L} p_l) \\ &\quad + \sum_{l=2}^{L} g^{L-l}(1-\epsilon)^l(\prod_{k=1}^{l} p_k) + G \\ &= c_0 + c_1 \sum_{l=1}^{L} p_l + \sum_{l=2}^{L} c_2^l \prod_{k=1}^{l} p_k + G\end{aligned} \tag{4}$$

where c_0, c_1, c_2 are scalar coefficients, and G is a placeholder for a large number of similar terms we were unable to express concisely. The terms in G all have a similar form in the sense they are all a product of three term, xyz, where x is a scalar, $y = \sum_{l=1}^{k} p_l$ is a sum of some number k probabilities, and $z = \prod_{k}^{L-n} p_k$ where n is some number less than L.

Thus, the joint may be expressed with four terms: A constant scalar c_0, a sum of the conditional probabilities $c_1 \sum_{l=0}^{L} p_l$, a product of probabilities, and a large sum of terms that multiply a sum of probabilities by a product of probabilities. Let's collapse G and the product term into the term S. In the limit where $S \to 0$ we have

$$\begin{aligned}\text{argmax}_{h,\theta}\, p(h_0^*, h_1^*, ..., h_L^*) &= \lim_{S \to 0} \text{argmax}_{h,\theta}\, c_0 + c_1 \sum_{l=0}^{L} p_l + S \\ &= \text{argmax}_{h,\theta}\, c_0 + c_1 \sum_{l=0}^{L} p_l \\ &= \text{argmax}_{h,\theta} \sum_{l=0}^{L} p_l \\ &= \text{argmax}_{h,\theta}\, E.\end{aligned} \tag{5}$$

Thus, under this limit $E \approx p(h_0^*, h_1^*, ..., h_L^*)$.

In what cases is this limit well approximated in practice? Each term in S has form xyz, where x is a non-zero scalar, y will typically be non-zero since it is a sum of probabilities, and z is a product of probabilities. The term z, therefore, will be zero when at least one term in the product is zeroed out. The more terms in the product z, the more possible terms there are that could set $z = 0$. Thus we can see that if at some training iteration, there are a significant number of learned conditional probabilities that are zeroed out, there will be a significant number of terms in S that are zeroed out too.

When will a significant number of learned conditional probabilities be zerod out? First, this will happen for all networks early in training, since neurons and there synapses must be grown one at a time. For example, if a node l grows a new neuron at the current iteration, t, its conditional probability according to the parameters at the current iteration will be zero. If node l takes a value that was not previously observed in combination with its sibling node values, it (or at least one of its siblings) will have probability 0. This effect should be amplified when nodes are allowed to grow more neurons, and when there are more nodes that could have a zero conditional probability. This suggests that in larger networks, especially early in training when α is large, maximizing our energy function in SQHNs closely approximates maximizing the joint with a Dirichlet prior over parameters.

1.2 Supplementary Note 2: Derivation of Inference Procedure

MAP inference in tree-structured graphs with discrete nodes can be implemented via the max-product algorithm [7, 1, 10], whose goal can be described as:

$$\textbf{Max-Product:}\ \text{argmax}_{h^*} \prod_{h_l^*} p(h_l^*|pa_l^t), \tag{6}$$

where h^* is the set of integer value assignments for each node. The max-product algorithm works by performing a single FF and FB sweep through the network. Consider a tree structured graph where, without loss of generality, we assume the simple case where nodes take one of two value. In this case, the conditional probability of node l given its parent node are represented by matrix $M_{l,pa_l} = [m_0, m_1]$. The max-product algorithm first propagates a signal from the visible nodes up through hidden nodes to the root node using the operation (in our notation) $h_{pa_l} = [\max(m_0 \otimes h_l), \max(m_1 \otimes h_l)]$ [1], where $\max$ (not to be confused with the activation function max) outputs a single scalar. In the case of multiple children nodes inputs to h_l are multiplied element-wise. The root node value is set equal to this maximum value, $h^*_L = \text{argmax}\, h_L$, then an operation known as backtracking is performed, where a signal is propagated back from the root node down the tree to find the MAP values for hidden nodes (see [1] for details). An analogous algorithm may be used to maximize the SQHN energy E, which is a sum rather than product of conditional probabilities. The analogous operation is $h_2 = [\max(m_0 + h_1), \max(m_1 + h_1)]$. These operations may be used in the same procedure as the max-product to obtain the max E value at the root node, MAP hidden node values via a backtracking procedure.

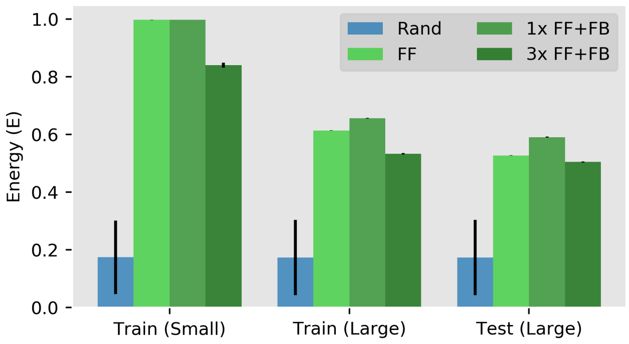

Supplementary Figure 1: Energy measurements during SQHN inference procedure. Energy after a single feed forward (FF) sweep, one FF and feedback sweep (1x FF+FB), and three FF FB sweep (3x FF+FB). Energy shown on small train set (200 images), and on large train set (1000 images) and test/hold-out data set. One FB sweep increases energy, but the network performs best only after a single FF and FB sweep.

The issue with this approach is that the main operation used in the FF sweep is not a standard vector matrix multiply. Instead of multiplying each row/memory vector of $M_2^\top$ element-wise by h_1 then summing over elements, as in a matrix multiply, each row and h_1 are multiplied element-wise then the max value is returned. However, our goal is to create a neural network, that may be easily implemented in hardware that assumes vector matrix multiplies as the main operation (e.g., GPUs and memoristor based neuromorphic hardware). Therefore, we propose the following alternative approximate MAP inference procedure, which only uses vector matrix multiplies and a neuron-wise normalization operation. Like the MAP inference procedures described above, this procedure involves a feed forward (FF) and feedback sweep through the network. During the FF sweep, each node, starting at the lowest layer and working up, updates according to:

$$h_l = \frac{1}{Z} \sum_{c \in ch(h_l)}^{C} M^\top_{l,c} max(h_c), \tag{7}$$

where $ch(l)$ is the set of nodes that are children of h_l and $M_{l,c}$ is the matrix from node l to child node c and the italicized max operation to express an activation function that output a vector of all zeros, except for the max element: e.g., $max([.3, .8, .4]) = [0, .8, 0]$. The summed input is normalized by $Z = \frac{1}{C\sqrt{\sum_c \max(h_c)^2}}$.

We can also describe the activation at l's child node as $max(h_c) = argmax(h_c) \max(h_c) = h^*_c \max(h_c)$, where $argmax$ is the activation that returns a one-hot, and the un-italized $\max$ returns a scalar, e.g., $\max([.3, .8, .4]) = .8$. Further, for each each neuron j in node l it is the case that $M^\top_{l,c,j} h^*_c = p(h^*_c | h_l = j)$. This means we can express the input to each neuron j in node l as

$$h_{l,j} = \frac{1}{Z} \sum_{c \in ch(h_l)}^{C} p(h^*_c | h_l = j) \max(h_c). \tag{8}$$

Thus, the input to each neuron j, during the FF sweep is a sum of the probabilities of child nodes, given node l has value j, where each probability is weighted by the associated max value at the child node. Therefore, $h_{l,j}$ is the weighted energy of l's children node values, when l takes value j. The term Z then takes the average, so that $h_{l,j}$ is a weighted average of the probabilities of child nodes given node l has value j. Thus, the FF sweep sets nodes to the value that maximizes the weighted average of the probability (i.e., weight energy) of child node values.

Importantly, the weighting terms carry information about the energy of descendent nodes: the weighting term $\max(h_c)$ can be defined recursively via equation 8, which shows that h_c is itself a weighted average of its children node probabilities (i.e., grandchildren of l), and so on. Therefore, weighting terms act as a kind of bottom up attention mechanism, where inputs from children are weighted based on the weighted energy/probability of grandchildren, whose weight are based on the energy/probability of great-grandchildren, and so on.

Nodes at the first hidden layer, on the other hand, receive a signal from visible nodes with no children. It turns out this signal can still be interpreted as the a weighted average of the likelihood of each pixel value. The input to a node at the first hidden layer is

$$h_{l,j} = \frac{.5(m_{l,j}^{\top} - .5)(x_{c_l} - .5)}{\|(m_{l,j}^{\top} - .5)\|\|(x_{c_l} - .5)\|} + .5, \tag{9}$$

where x_{c_l} is a vector of input value (e.g., from an image patch). The input we use has values between 0 and 1. We treat each value as a binary variable whose probability is

$$\begin{aligned} p(x_{c_l,i}|h_l^*) &= x_{c_l,i}p_{c_l,i} + (1 - x_{c_l,i})(1 - p_{c_l,i}) \\ &= 2x_{c_l,i}p_{c_l,i} - x_{c_l,i} - p_{c_l,i} + 1 \end{aligned} \tag{10}$$

Next, consider that the prediction p_{c_l} just equals the memory vector $m_{l,j}$ indexed by h_l^*. Given this, we can rewrite the numerator of equation 9 as

$$\begin{aligned} (m_{l,j,i} - .5)(x_{c_l,i} - .5) &= (p_{c_l,i} - .5)(x_{c_l,i} - .5) \\ &= p_{c_l,i}x_{c_l,i} - .5p_{c_l,i} - .5x_{c_l,i} + .25 \\ &= .5p(x_{c_l,i}|h_l^*) - .25, \end{aligned} \tag{11}$$

which is just the liklihood of the input with an affine shift applied. Thus, the numerator in equation 9 computes an affine shifted version of the likelihood of each pixel and sums these together. The denominator ensures the output is between -1 and 1, which is rescaled to the range 0 and 1 by the multiplication and shift of .5. The advantage of computing the likelihood this way, rather than a direct computation of equation 10 is that this operation can be implemented via a neural network like matrix vector multiply with neuron-wise normalization:

$$h_l = \frac{1}{2Z}(M_l^{\top} - .5)(x_{c_l} - .5) + .5, \tag{12}$$

where Z is a vector of the terms computed accord to the denominator in equation 9. It is also possible to just store a separate feedback matrix, with shifted and normalized rows.

Finally, after activities h_l are computed via the FF sweep, the final h_l values are computed using the top-down/FB sweep. Let h_{l+n} be the parent of h_l

$$h_l = (\lambda)h_l + (1 - \lambda)p_l, \tag{13}$$

where $p_l = M_{pa_l,l}h_{pa_l}^*$ and λ is a scalar between 0 and 1 that modulates the influence of the top-down signal, which we typically set to .5 during recall.

Supplementary Algorithm 1: SQHN Recall Algorithm

begin
 for $t = 1$ **to** T **do**
  // Clamp visible nodes to x^t
  // Inference
  for $l = 0$ **to** L **do**
   // Compute bottom up input h_l, equation 9, 7
  end
  for *reversed(*$l = 0$ **to** $L - 1$*)* **do**
   // Combine h_l and top-down input, equation 13
   // Set $h_l^* = argmax(h_l)$
  end
  // Set each input patch $x_{c_l} = Mh_l^*$
 end
end

1.3 Suplementary Note 3: Neuron Growth and the Dirichlet Process Prior

The inference process described above assumes a fixed number of neurons at each node. It is assumed neuron numbers are fixed during recall, when we are performing inference over old data points, from the training set. However, intuitively, it may be desirable to add new neurons and synapses dynamically as needed during learning, when the model performs inference over new data points. A common principled way to dynamically add new components to a discrete/categorical distribution is via the Dirichlet process prior. In parametric models with discrete variables (e.g., standard mixture models), the number of

components J is treated as a hyper-parameter, which set by the modeler. In non-parameteric models, J is learned/inferred by the model according to a prior distribution over J. The Dirichlet process prior (DPP) is a common prior for discrete non-parameteric models. We do not go into details on the derivation of the DPP prior here. We only explain how it inspires our model. For good tutorials see [4, 6].

The DPP provides a method for determining when it is more probable that some input belongs to a new latent, integer value $(J + 1)$, rather than an already existing value $(\leq J)$. It does this by representing the prior probability that the input belongs to a new integer value, given all previous assignments: $p(h_l^{*,t} = new|h_l^{*,0:t-1})$, which is the prior probability the input to node l at iteration t belongs to a new value given all of the previous value assignments up until current time t. The DPP sets this prior probability as

$$p(h_l^{*,t} = new|h_l^{*,0:t-1}, \alpha) = \frac{\alpha}{t + 1 + \alpha} \tag{14}$$

where α is a hyper-parameter. The DPP is order invariant [4], which means that the ordering the previous data points arrive does not affect the computation of the prior. The posterior probability that the node value is new, multiplies the DPP above with the likelihood of the input according to some prior distribution over parameters [6]. This posterior can then be compared to the posterior probabilities of existing values given a data point, x, and existing parameters. One can then make the decision to add a new value, if this is the most probable case.

Since the SQHN maximizes an energy function rather than the actual joint distribution and performs approximate inference rather than exact MAP inferance over the energy, our algorithm for neuron growth is inspired by the DPP rather than an exact implementation of it. In particular, we use the following simple computation as an estimate of the energy value associated with adding a new neuron:

$$\epsilon = \frac{\gamma\alpha}{t + 1 + \alpha}, \tag{15}$$

where α and γ are hyper-parameters and $0 < \gamma \leq 1$ and $0 < \alpha$. One can think about γ as analogous to an estimate of the likelihood the input belongs to a new value (according to some prior distribution) and $\frac{\alpha}{t+1+\alpha}$ as analogous to the prior probability of the the input belonging to a new node value/neuron. During learning the SQHN performs inference before updating weights. If the maximum internal state value (energy), $\max(h_l)$, at node l is less than ϵ, a new neuron is grown. Otherwise, the node is set to the maximum existing value (see next section).

1.4 Supplementary Note 4: Derivation of Learning Algorithm

Under the SQHN algorithm, parameters are updated each iteration to solve the following optimization problem:

$$\begin{aligned} \theta^t &= \text{argmax}_\theta \sum_{i=0}^{t} E(\theta, h^{*,i}, x^i) \\ \text{s.t.} &\sum_j p_{l,j} = 1 \; \forall l^{hid} \text{ and } p_{l,j} \geq 0 \; \forall l, j \end{aligned} \tag{16}$$

where $h^{*,i}$ are one-hot value assignments for each node associated with data point x^i, t is the current training iteration, x^i is the data point presented at iteration i, and l^{hid} refers to hidden nodes. The weight update at iteration t sets parameters equal to the values that maximize the energy over the current and all previously observed data points, and associative hidden states, under the constraint the prediction p_l at hidden nodes are properly normalized. Importantly, since this update is computed online, we assume we have to perform this update only provided the data point and hidden states at the current iteration (i.e., we are assuming there is no buffer storing previous data and hidden states). This leaves two questions: 1) How do we solve this constrained optimization problem, in the online setting, and 2) how should the values of h^* be set each iteration?

The same optimization above has been solved for the Bayesian networks [5]. Bayesian networks update parameters to maximize the product of conditional probabilities. SQHN networks update parameters to maximize the sum of conditional probabilities. Despite these differences, the goal ends up being equivalent w.r.t. to parameter updates: each matrix representing the conditional probabilities between parents and children must be updated to maximize the conditional probabilities of the observed child values (under normalization constraints). Thus, SQHN networks can utilize the solution for Bayesian networks as a basis for its online learning rule.

Consider the case where child node, l, is a discrete/categorical variable. The matrix containing the conditional probabilities between the parent of l, pa_l and l is M_{l,pa_l}. The columns of this matrix contain the conditional probability distributions given various values of the parent node: $m_{l,pa_l} = [m_{l,0}, m_{l,1}..., m_{l,J}]$, where $m_{l,j}$ is the conditional probability distribution over values of l, given $pa_l = j$. Each value of $m_{l,j}$ tells us the probability that l takes a particular value given the $pa_l = j$. In particular, $p(l = k|pa_l = j) = m_{l,j,k}$, where k is the kth value of $m_{l,j}$. The update for Bayesian networks tells us that this

value is simply the number of times j and k co-occurred previously, divided by the total number of times parent to value j occurred [5]:

$$p(l = k|pa_l = j) = m_{l,j,k} = \frac{c_{l,j,k}}{c_{l,j}}, \tag{17}$$

where $c_{l,j,k}$ is the number of times $pa_l = j$ and $l = k$ at the same iteration. The term $c_{l,j}$ is the number of times $pa_l = j$. This solution entails the matrix column $m_{l,j}$ is an average over the one-hot values of the child node that were present when $pa_l = j$:

$$m^t_{l,j} = \frac{1}{c^t_{l,j}} \sum_{i \in t_j} h^{*,i}_l, \tag{18}$$

where t_j refers to the set of time steps where $pa_l = j$. This same average can be computed online:

$$m^t_{l,j} = m^{t-1}_{l,j} + \frac{1}{c^t_{l,j}}(h^{*,t}_l - m^{t-1}_{l,j}), \tag{19}$$

under the assumption $pa_l = j$. This same update can more generally be implemented as a local, Hebbian-like learning rule:

$$M^t_l = M^{t-1}_l + \frac{1}{c^{t,\top}_l h^*_{pa_l}}(h^{*,t}_l - p^{t-1}_l)h^{*,t\top}_{pa_l} \tag{20}$$

where the update uses the fact that $p_l = M_l h^*_{pa_l}$ which trivially entails that p_l equals the memory vector indexed by $h^*_{pa_l}$, e.g., if $pa_l = j$ then $p_l = m_{l,j}$. Second, the step size $\frac{1}{c^{t,\top}_l h^*_{pa_l}}$ uses the fact that $h^*_{pa_l}$ indexes the correct count, e.g. if $pa_l = j$ then $c^{t,\top}_l h^*_{pa_l} = c^t_{l,j}$.

This is the update rule if children nodes are are discrete/categorical variables. We use the same update for visible nodes, which we treat as vectors of binary variables. The properties remain the same in this case: $m_{l,j,k}$ ends up representing an average over previously observed values of input value k in image patch l.

Next, is the question of how to set the hidden node values during training? The weight updates above assume h^*_l values are observed. However, when h^*_l is a hidden node its values are, by definition, not observed. One option for setting hidden node values is to use MAP learning. MAP learning works by first performing MAP inference over hidden variables to find the specific values of the hidden variables, h^*, that maximize the posterior $P(h^*|x, \theta) \propto P(h^*, x, \theta)$. Parameters are then updated to further increase the probability of the joint $P(h^*, x, \theta)$. We use a variant of MAP learning here. In particular, hidden node values are determined by an inference procedure where each node maximizes the probability of its children nodes: each node receives the FF signal from its children, then it is either assigned a one-hot indexing the existing neuron with the maximum internal state value, or if all existing neuron values are below the threshold ϵ, the node is set to a new value (i.e., a new neuron is grown) (see algorithm 2).

The resulting algorithm as a kind of hybrid between MAP learning and learning in the case where all node values are observed. Like MAP learning we set the hidden variables to a point estimate value with the highest probability. (This is opposed to computing a distribution over hidden nodes values, which is what the common expectation maximization [2, 5]) algorithm does.) However, unlike MAP learning, we do not compute the maximum of the posterior since we ignore FB/top-down signal in inference. Instead, similar to [3], our algorithm computes the values that maximize the conditional probability of its child node values, while ignoring parent node values. Nodes, therefore, maximize the likelihood of their child node values. Thus, like the case where all node values are observed, each parent node does not influence/determine the value of its child node, but instead only 'observes' its child's values and updates its conditional distribution accordingly. But unlike this fully observed case, hidden node values are set via inference.

We find this max-likelihood variant works better than MAP in the online setting. MAP inference ends up pushing hidden node values to the values that were inferred in previous iterations, making the model get 'stuck' reproducing the same hidden variable values rather than learning new combinations of values for different inputs. Remov-

ing the FB/top-down influence during learning allows nodes to assign new sets of values to new inputs, as desired.

Supplementary Algorithm 2: SQHN Learning Algorithm

```
begin
    for t = 1 to T do
        // Clamp visible nodes to x^t
        // Max Likelihood Inference
        for l = 0 to L do
            // Compute bottom up input h_l, equation 7, 9
            // If h_l is less than ε, set h*_l to new value.
            // Else h*_l = argmax(h_l)
        end
        // Update ε, equation 15
        // Update Weights, equation 20
    end
end
```

1.5 Supplementary Note 5: Derivation of Episodic Recognition Rule

Recognition tasks begin by presenting a sequence of items/data points from the training set X_{train} during a training phase. Then during a testing phase, the model is presented with a mixture of old data points from X_{train} and new data points from a similar (in-distribution) data set $X_{in-dist.}$ and from a dissimilar (out of distribution) data set $X_{out-dist.}$. An equal portion of data is presented from each data set during testing. The model must correctly judge if the data point is in the training set (old) or not (new). This task is based on classic and common tests of human memory [8].

Importantly, episodic recognition is distinct from the common OOD detection task in machine learning and will therefore require a different solution. OOD detection is the task of detecting data drawn from a distribution distinct from the training set [11]. Solutions typically involve learning a generative model of the training data, then computing the likelihood of data under this model [11]. This is not the same as detecting data that was not present during training, since many unobserved/new data may still be from sampled from the same distribution as the training set, yet still be unobserved/new.

Inspired by probabilistic models of human episodic recognition (e.g. [9]), we instead use the following approach. We assume each training input x^t is mapped to, what we call, a global feature representation h^*. By global, we mean it represents features of the entire input (e.g., entire image) rather than just a sub-portion of it (e.g. image patch). Then each feature vector is stored in an itemized memory via a mixture model. Let M be the matrix that contains cluster means. Feature representations, $h^{*,t}$ at each training iteration t, get stored in the columns of M, where $M = [h^{*,0}, h^{*,1}, ...]$. If every data point from the train set gets stored separately then during testing the model can simply compare a feature representation h^* of the input to the stored feature representations. If the likelihood is around 1, then the model judges 'old' if less then one, it judges new.

Up until it reaches capacity, and with a high α, this is exactly what happens at the memory node of SQHN. The memory node has full receptive field and takes as input the activities from the values of its children, which represent the input's visual features. Thus, until capacity is reached, SQHN has a clear way of making old/new judgments: perform max-likelihood inference (see algorithm 2), and check if children of the memory node have probability/energy ≈ 1.

However, after capacity is reached, every data point is not stored in M_L in its original form. Instead, new inputs will be averaged with old ones. This raises the question of how judgments should be make after capacity is reached. We use the following strategy. Let $p(h^*_{c_L}|h^*_L = j)$ be the likelihood of the values of the children of the memory node given the memory node value $h^*_L = j$. Here for simplicity we assume this is a good estimate of the likelihood $p(x^t|h^*_L = j)$. Let's say n data points from the train set have been assigned to j. Lets say we know the data point assigned to this value with the lowest likelihood is x_{min}. If we know this likelihood, we know any new data point assigned to j that has a likelihood lower than this minimum must be new (i.e., have 0 probability of being old). Thus, we propose approximating the probability that some data point x^t is old using the re-scaled likelihood

$$p(x^t = old|h^*_L = j) \approx p(x^t|h^*_L = j) * (1 - p(x_{min}|h^*_L = j)) + p(x_{min}|h^*_L = j) \quad (21)$$

Then to make a recognition judgments, the model checks if $p(x^t = old|h^*_L = j)$ is greater than or less than .5. Equivalently, we could set a threshold, μ, equal to the likelihood value at which $p(x^t = old|h^*_L = j) = .5$. In practice, we find the minimum and midpoint likelihood difficult to exactly compute online. Instead, we use a simple estimate of the mid-point

value, which we find works well in practice. This estimate just keeps a moving average of the likelihoods:

$$\mu^t_{L,j} = \frac{c^t_{L,j} - 1}{c^t_{L,j}} \mu^{t-1}_{L,j} + \frac{1}{c^t_{L,j}} h^t_{L,j}, \tag{22}$$

where j is the neuron with the maximum value, $h_{L,j}$, at root node L at iteration t.

Supplementary Algorithm 3: SQHN Episodic Recognition Algorithm

```
begin
    for t = 1 to T do
        // Clamp visible nodes to x^t
        // Max Likelihood Inference (w/o Neuron Growth)
        for l = 0 to L do
            // Compute bottom up input h_l, equation 9, 7
            h*_l = argmax(h_l)
        end
        // If max(h_L) > μ_j (where argmax(h_L) = j), judge old
        // Else judge new
    end
end
```

1.6 Supplementary Note 6: Theoretical Results

Capacity. We analyze the properties of the recall accuracy of an SQHN with one hidden layer. We define the capacity of the network as the maximum number of data points the network is able to recall given some recall threshold γ. Let J be the number of neurons at the hidden layer.

Theorem 1. *Assume no two data points in the training data set are scalar multiples of each other and during recall data points are not corrupted. The capacity of a single hidden layer SQHN network is at least J, i.e., the number of neurons at the hidden layer, for any $\gamma \geq 0$.*

Proof. Assume a single data point is stored in each memory vector. If the child node is a discrete variable then the input to each hidden neuron, j is the cosine similarity between the input and the jth memory vector. If the child node is a binary variable then the input to each hidden neuron, j is the mean-shifted cosine similarity (see main article equation 14) between the input and the jth memory vector. In either case, the vector with the maximum similarity value will be returned, as long as no two vectors have the same angle. We assume no to vectors have the same angle (no two stored vectors are scalar multiples of each other). Therefore, the maximum value at the hidden layer is guaranteed to index the correct stored training data point, which will yield perfectly accurate MSE of 0 and thus perfect recall accuracy for any γ. If some memory vectors average over data points, it is not guaranteed to yield correct recall though this is possible, for some values of γ and under certain distributions of training data for obvious reasons. Thus, the minimum capacity is at least the number of neurons at the hidden layer, J. □

Forgetting. It is also important to understand what happens when the SQHN network is pushed passed capacity. Ideally, memory models should show 'graceful' forgetting in such cases, where performance decreases in a predictable and non-abrupt manner. We assume a kind of worst-case conditions where 1) data points are highly orthogonal such that averaging two or more data points in some memory vector m_j means that returning the memory vector m_j during recall does meet the condition for successful recall for any of the data points it was averaged over, and 2) each existing memory vector is equally likely to be accessed and updated during training.

Theorem 2. *Under the worst case assumptions, the number of memory vectors that store a single data point decreases at an exponential rate of $Je^{\frac{-t}{J}}$, where t is the training iteration (starting after the network is at capacity) and J is the number of hidden units. This entails its recall accuracy will decay at rate $\frac{Je^{\frac{-t}{J}}}{J+t}$.*

Proof. The process we are characterizing is the training of a basic SQHN unit that begins at capacity (each memory vector is set equal to one previously observed data point). Each training iteration, t, a memory vector is chosen with uniform probability and the data point observed at that iteration is averaged with the chosen memory vector. There are thus two kinds of memory vectors: those that store a single data point and those that store an average over more than one data point. We want to characterize how the number of memory vectors that store only a single data point decrease over time on average, where by 'on average' we mean that the decreases are measured and averaged over an infinite number of training runs.

Let $I(t)$ be the number of memory vectors, at time t, that store only a single data point. Under these assumptions, the decrease in $I(t)$ is clearly equal to the probability of choosing and updating a memory vector that store only a single data point, since this is equal to the proportion of training runs that will choose and update such a memory vector. Under the assumption of a uniform probability distribution, the probability of choosing and updating a memory vector that equals a single data point is just equal to the proportion of memories that store a single data point at time t:

$$\frac{\partial I(t)}{\partial t} = -\frac{I(t)}{N}. \tag{23}$$

A function $f(x)$ decays exponentially according to $f(x_0)e^{-\lambda x}$ with rate λ and initial value $f(x_0)$, if $\frac{\partial f(x)}{\partial x} = -\lambda f(x)$. Clearly, the rate of forgetting shown above is an exponential with rate $\frac{1}{N}$. At the initial iteration it is assumed $I(0) = N$. Therefore, the forget rate can be described as $Ne^{\frac{-t}{N}}$. □

Thus, the SQHN memory unit or one hidden layer model shows a graceful, exponentially decaying forget rate.

Learning as Proximal Parameter Isolation. Let's consider a distance metric between the parameters before and after the update in terms of the amount of energy that is left for parameters to reduce:

$$d(M_l^{new}, M_l^{old}) = E_{ch_l}(M_l^{new}, h_l^*, h_{ch_l}^*) - E_{ch_l}(M_l^{old}, h_l^*, h_{ch_l}^*), \tag{24}$$

where E_{ch_l} is the energy (averaged conditional probability) of the children of l, and M_l^{old} is the matrix of conditional probabilities from l to its children before the weight update, and M_l^{new} the conditional probabilities after. Thus, this distance measure tells us how different the conditional probabilities of the children are under the new and old matrix.

Here we analyze properties of the SQHN learning algorithm in a case where two simplifying assumptions are made:

1. Each column of M_l is updated with an equal, constant step size.
2. The weight are updated to maximize energy to a specific value: $\kappa = E_{ch_l}(M_l^{new}, h_l^*, h_{ch_l}^*)$.

From this the following theorem clearly follows.

Theorem 3. *Under the assumptions above, the value of h_l^* computed during the training phase of the SQHN is equivalent to*

$$h_l^* = \text{argmin}_{h_l^*}\, d(M_l^{new}, M_l^{old}). \tag{25}$$

Proof. During learning, each node l takes value h_l^* that maximizes the energy of its children. Thus, during learning h_l^* is the value that maximizes $E_{ch_l}(M_l^{old}, h_l^*, h_{ch_l}^*)$. From assumption 2, we know after the update for any value of h_l^*, the energy of the children is κ. Thus, it clearly follows that the value h_l^* can be described as the value minimizing

$$\begin{aligned} \kappa - E_{ch_l}(M_l^{old}, h_l^*, h_{ch_l}^*) &= E_{ch_l}(M_l^{new}, h_l^*, h_{ch_l}^*) - E_{ch_l}(M_l^{old}, h_l^*, h_{ch_l}^*) \\ &= d(M_l^{new}, M_l^{old}). \end{aligned} \tag{26}$$

□

In this simplified case, setting h_l^* to the value that maximizes the energy of the children of l, is equivalent to setting h_l^* equal to the value that minimizes the amount that conditional probabilities encoded in M_l change during the weight update. By first maximizing the energy w.r.t. h_l^*, there is less 'work' for weight updates to do when they increase energy w.r.t. M_l. In other words, the SQHN algorithm seems to have a principled method for choosing which parameters to isolation during training, i.e., update the column in M_l that requires the least amount of change given the value of children nodes. This makes sense from the point of view of prevent catastrophic forgetting, where we generally want to reduce the amount parameters change. Future work could look to understand how this result applies to less simplified cases, better describing the SQHN.

1.7 Supplementary Note 7: Further Experiments

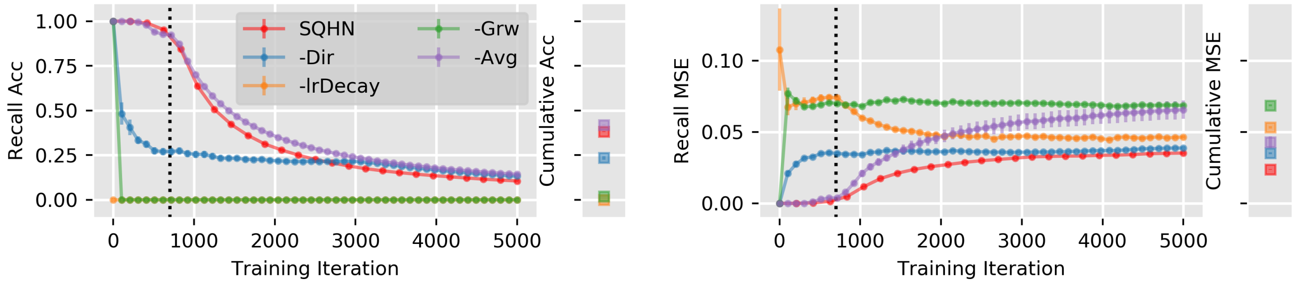

Supplementary Figure 2: Ablation study. We test the effects of removing various components of the SQHN learning algorithm in the online continual recall task on CIFAR-100 for the basic single hidden layer SQHN unit. We test the following ablations: 1) remove the Dirichlet based, exponentially decaying grow threshold and replace with a constant threshold (-Dir). 2) Remove the learning rate decay schedule and replace with a constant learning rate (-lrDecay). 3) Remove the grow operation and instead randomly initialize weights (-Grw). 4) Remove the averaging update and only grow new memory vectors (-Avg). The decaying grow threshold, learning rate schedule, and the grow operation are essential for high performance before the capacity (vertical dotted line) is reached. The grow operation, learning rate decay, and averaging operation are all essential for high performance after capacity is reached. Note that removing averaging helped slightly with the number of recalled images, however, doing so significantly worsened recall MSE. All components are necessary for a high cumulative performance under both measures.Recall accuracy and cumulative accuracy for various ablations is shown on the left. Recall MSE and cumulative MSE for the same ablations shown on right.

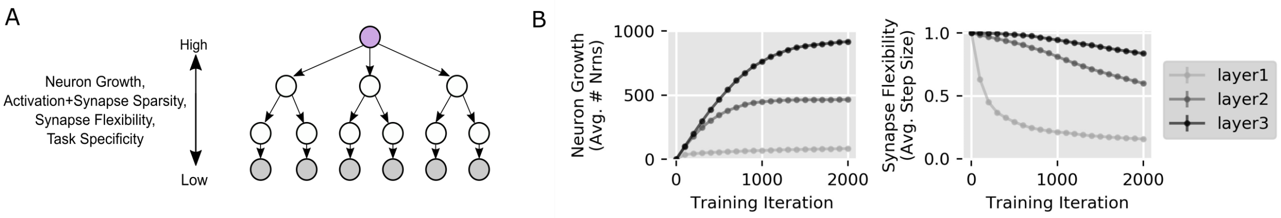

Supplementary Figure 3: Emergent properties. **A** Diagram summary of the emergent properties. **B** Measurements of neuron growth (left) and synapse flexibility (right) at each layer during training in online scenario in an SQHN with three hidden layers. Each node in the network has the same maximum number of neurons (1000), the same grow threshold, the same input kernal size (4x4), same learning rate decay, etc. The only difference between each node is its location in the network.

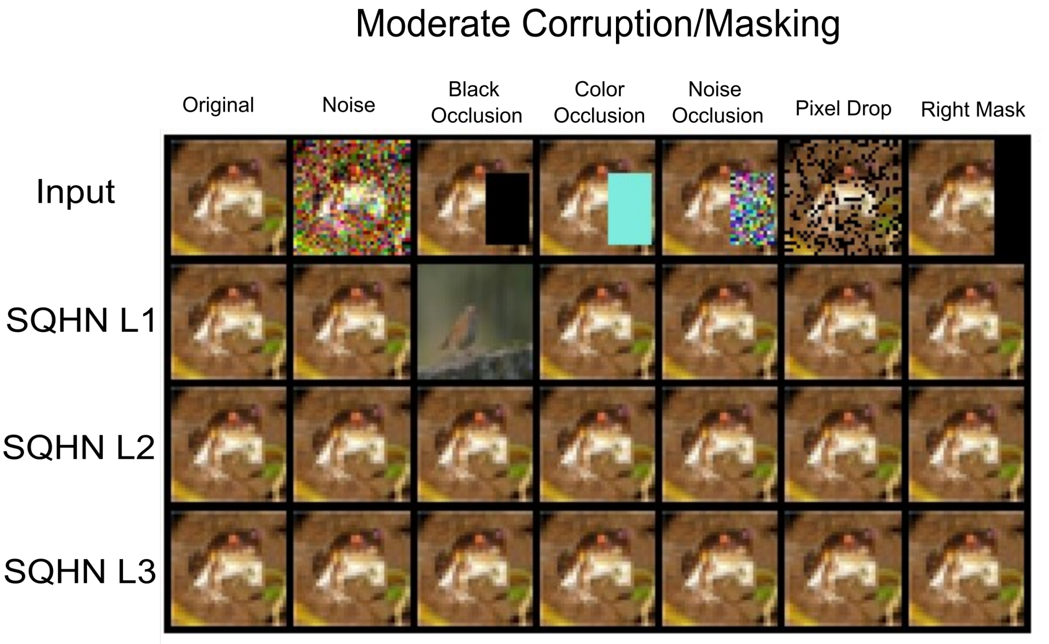

Supplementary Figure 4: Example outputs for moderate corruption scenario. Noise variance .2, masking and occlusion covers .25 of image. Trees are better at occlusion, but otherwise, with moderate corruption/masking all networks perform very well on all tasks.

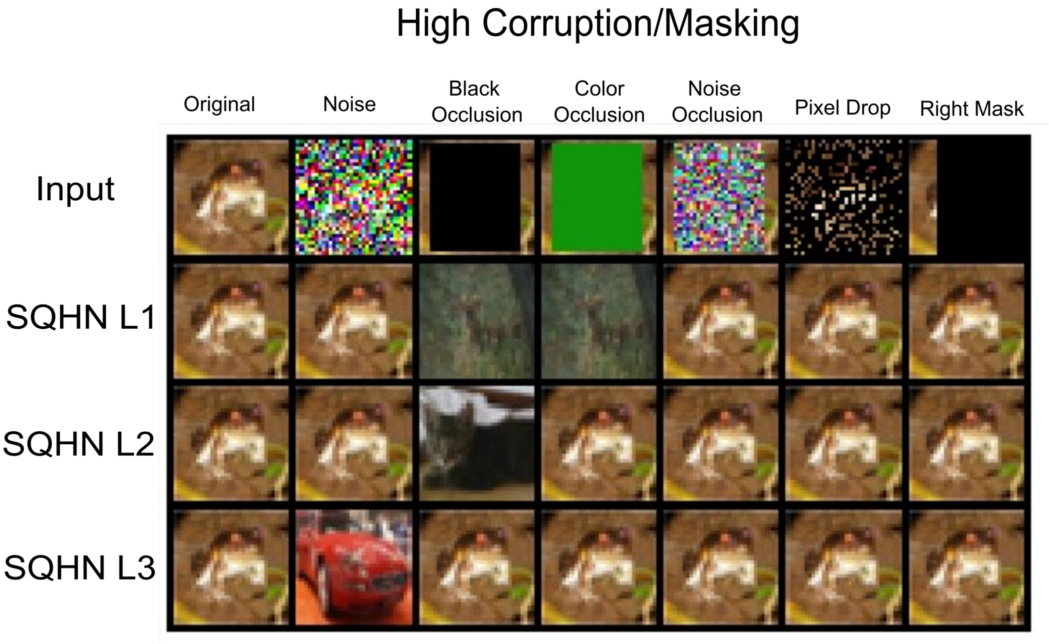

Supplementary Figure 5: Example outputs for high corruption scenario. Noise variance .8, masking and occlusion covers .75 of image. Taller trees, especially L3, are better at occlusion, but because they have small receptive fields at bottom layers they are more sensitive to noise. Smaller receptive fields mean smaller input vector per node, and smaller vector are more likely to overlap due to noise. The part-whole representation of the image, however, allows trees with multiple levels to better ignore occluded regions of the image.

Supplementary References

[1] Christopher M Bishop and Nasser M Nasrabadi. *Pattern recognition and machine learning*, volume 4. Springer, 2006.

[2] Arthur P Dempster, Nan M Laird, and Donald B Rubin. Maximum likelihood from incomplete data via the em algorithm. *Journal of the royal statistical society: series B (methodological)*, 39(1):1–22, 1977.

[3] Dileep George, Wolfgang Lehrach, Ken Kansky, Miguel Lázaro-Gredilla, Christopher Laan, Bhaskara Marthi, Xinghua Lou, Zhaoshi Meng, Yi Liu, Huayan Wang, et al. A generative vision model that trains with high data efficiency and breaks text-based captchas. *Science*, 358(6368):eaag2612, 2017.

[4] Samuel J Gershman and David M Blei. A tutorial on bayesian nonparametric models. *Journal of Mathematical Psychology*, 56(1):1–12, 2012.

[5] David Heckerman. *A tutorial on learning with Bayesian networks*. Springer, 1998.

[6] Yuelin Li, Elizabeth Schofield, and Mithat Gönen. A tutorial on dirichlet process mixture modeling. *Journal of mathematical psychology*, 91:128–144, 2019.

[7] Judea Pearl. *Probabilistic reasoning in intelligent systems: networks of plausible inference*. Morgan kaufmann, 1988.

[8] Michael D Rugg and Andrew P Yonelinas. Human recognition memory: a cognitive neuroscience perspective. *Trends in cognitive sciences*, 7(7):313–319, 2003.

[9] Richard M Shiffrin and Mark Steyvers. A model for recognition memory: Rem—retrieving effectively from memory. *Psychonomic bulletin & review*, 4:145–166, 1997.

[10] Yair Weiss and William T Freeman. On the optimality of solutions of the max-product belief-propagation algorithm in arbitrary graphs. *IEEE Transactions on Information Theory*, 47(2):736–744, 2001.

[11] Jingkang Yang, Kaiyang Zhou, Yixuan Li, and Ziwei Liu. Generalized out-of-distribution detection: A survey. *arXiv preprint arXiv:2110.11334*, 2021.
